# Acute Primary Cutaneous Nocardiosis

**DOI:** 10.4269/ajtmh.23-0584

**Published:** 2024-03-26

**Authors:** Xiaokang Xu, Zehu Liu, Xiujiao Xia

**Affiliations:** ^1^ Changxing Skin Disease Hospital, Changxing County, Zhejiang, China;; ^2^ Department of Dermatology, Hangzhou Third People’s Hospital, Hangzhou Third Hospital Affiliated to Zhejiang Chinese Medical University, Hangzhou, China

**Figure 1.**
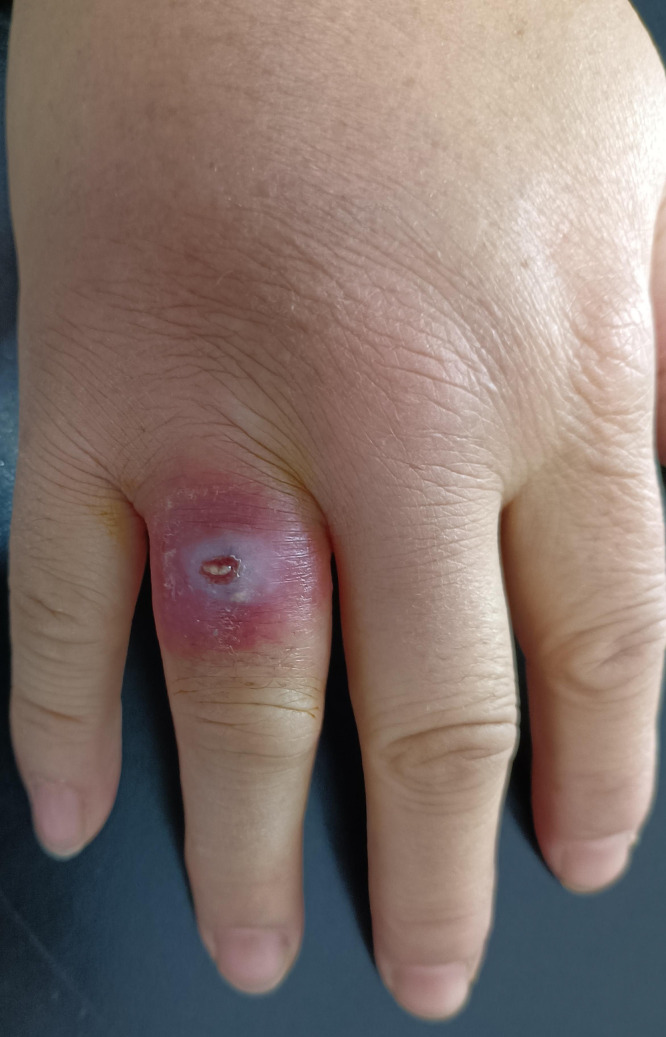
Erythematous swelling with a central ulcer on the root of the right ring finger.

**Figure 2.**
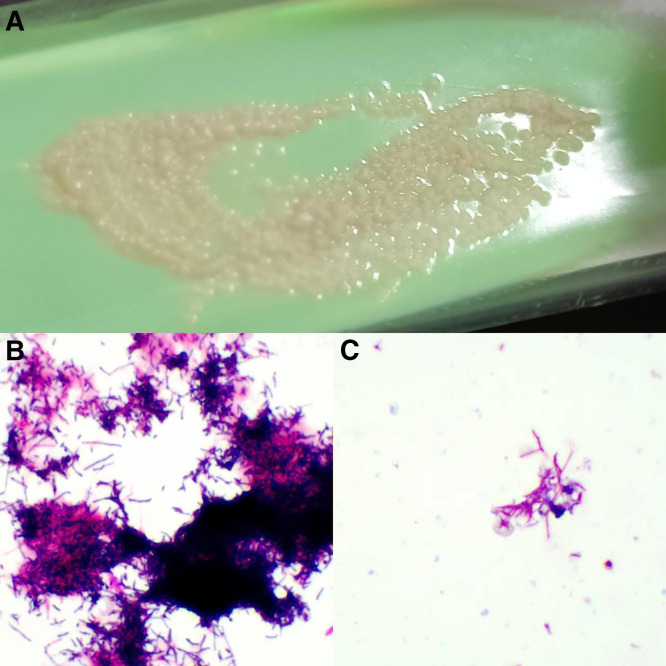
(**A**) Colonies on the Lowenstein–Jensen solid medium after 6 days of incubation at 25°C. (**B**) Gram staining of colonies (×1,000) and (**C**) Ziehl–Neelsen staining of colonies (×1,000).

**Figure 3.**
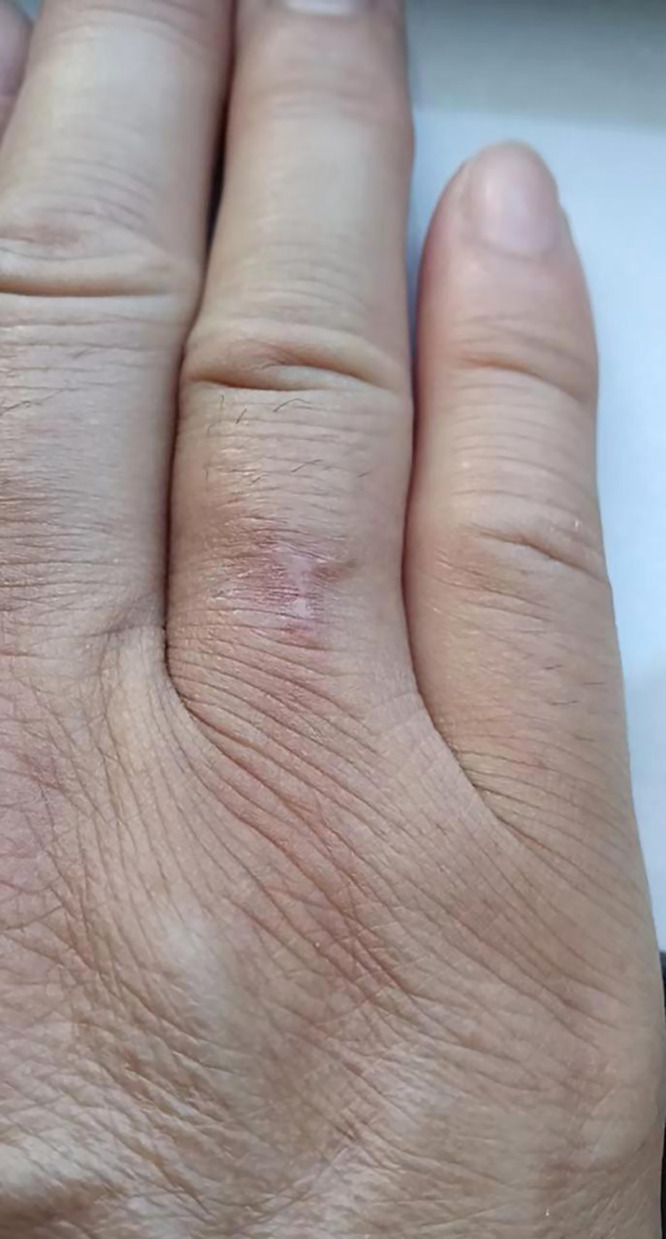
Complete healing of the lesion after treatment.

A 51-year-old woman came to our dermatology clinic with a swollen, slightly painful, ulcerative eruption on her right ring finger on 1 week’s duration. She first noticed a pustule arising on the dorsal aspect of her right finger without an obvious predisposing factor and picked out the pustule. A few days before presentation, she made steamed buns with flour and yeast powder. An erythematous lesion quickly developed around the pustule and then ruptured. Physical examination revealed a tender erythematous swelling with a 0.3 × 0.4 cm^2^ central ulcer on the root of the right ring finger (Figure 1). Ipsilateral axillary lymphadenectasis with tenderness was also palpable. The cardiovascular, pulmonary, and neurologic examinations were unremarkable. No underlying diseases causing immunocompromise, such as HIV, were present. A complete blood count showed a white blood cell of 11.5 × 10^9^/L with 78.2% neutrophils. We performed nontuberculous mycobacteria and fungal cultures on the pus from the ulcerative lesion. The results of fungal culture were negative, and pus culture on Lowenstein–Jensen medium at 25°C showed large, yellowish, raised, furrowed, moist colonies after 6 days of incubation (Figure 2A). Stain from pus culture smears were weakly Gram positive and partially acid-fast with a filamentous appearance (Figure 2B and C). Gene identification by 16S ribosomal RNA (16S rRNA) sequencing revealed *Nocardia brasiliensis* (GenBank accession number: OM919545). Disk diffusion susceptibility testing disclosed that the organism was sensitive to minocycline, ceftriaxone, amikacin, amoxicillin/clavulanic acid, linezolid, tobramycin, and sulfamethoxazole-trimethoprim but resistant to levofloxacin and imipenem. Treatment began with drip infusion of 2 g/day ceftriaxone. After 7 days of treatment, the lymphangitis and skin lesion had almost completely resolved. There was no recurrence after 6 months of follow-up (Figure 3).

Primary cutaneous nocardiosis is mainly caused by *Nocardia asteroides* and *N. brasiliensis*, with *N. brasiliensis* isolated in most cases of lymphocutaneous nocardiosis.^1^ Sometimes the clinical presentation of the disease resembles that of nontuberculous mycobacteria infections, such as *Mycobacterium marinum*. In this case, the early morphology of colonies on the Lowenstein–Jensen medium was similar to that of *M. marinum* colonies. Both bacteria usually appear acid-fast by Ziehl–Neelsen staining and Gram positive. Molecular typing is important for definitive identification.^2^

Cutaneous nocardiosis is mainly acquired through contact exposure to *Nocardia* in the environment.^3^ Patient exposure to flour and yeast powder after picking out the pustule may be the route of transmission. The first-line drugs for treating nocardiosis are sulfonamides. Nonsulfonamides are effective treatments for patients with primary cutaneous nocardiosis who are resistant or intolerant to sulfonamides.^4^ Among them, ceftriaxone and imipenem are the second-line treatment choices.^5^ This case suggests that intravenous ceftriaxone is an effective treatment for acute primary cutaneous nocardiosis.
